# Ultrafast Cine cardiac MRI in the assessment of left ventricular diastology

**DOI:** 10.1186/1532-429X-13-S1-P342

**Published:** 2011-02-02

**Authors:** Jeremy D Collins, Peter Weale, Neil Chatterjee, Marie Wasielewski, Sanjiv Shah, James Carr

**Affiliations:** 1 Northwestern University, Chicago, IL, USA

## Introduction

Cardiac MRI is the accepted gold standard for the assessment of left ventricular systolic function. However, no standards exist for the MRI assessment of left ventricular diastolic function. We hypothesize that the superior morphologic assessment enabled by ultrafast ECG gated cine TrueFISP imaging will enable assessment of left ventricular diastolic function.

## Purpose

The purpose of this study is to determine if morphologic assessment of the left atrial size coupled with assessment of left ventricular filling on ultrafast cine TrueFISP imaging is able to differentiate subjects with normal and abnormal left ventricular diastolic function.

## Methods

18 self-reported healthy volunteers (12 men, average age 35 years) and 6 patients with diastolic dysfunction (4 men, average age 52 years) underwent cardiac MRI on an investigational 1.5T cardiovascular magnet (Aera, Siemens Medical Solutions, Erlangen Germany). All subjects underwent ultrafast cine MR imaging in four and two chamber orientations (nominal TR 5.5 - 15.3 msec, heart rate dependent) using an ECG-gated parallel imaging accelerated steady state pulse sequence, optimized to minimize the temporal resolution. Atrial volumes were calculated using the area-length method during left ventricular isovolumetric relaxation. Myocardial relaxation was determined visually off four chamber cine images. Diastolic dysfunction was defined as present when myocardial relaxation was abnormal or left atrial size was increased in the absence of overt mitral valvular disease or fluid overloaded status. A single reviewer, blinded to subject identity reviewed all images and performed the above measurements. All healthy volunteers were assumed to have normal diastolic function; echocardiography with tissue Doppler was the gold standard in all patients.

## Results

The left ventricular diastolic function of 15 of 18 healthy volunteers (83%) and 5 of 6 patients (83%) was correctly classified at MRI. Three volunteers demonstrated borderline left atrial volume with sluggish chamber filling suggestive of diastolic dysfunction. There was a statistically significant difference in age between these three volunteers and the rest of the volunteer cohort (p<0.05). The average left atrial volume of healthy volunteers was not significantly different from that in patients with diastolic dysfunction (p>0.05). One patient with echocardiographic evidence of diastolic dysfunction was indeterminate by MRI secondary to moderate mitral regurgitation.

## Conclusions

Gated ultrafast cine Cardiac MRI may be able to distinguish subjects with diastolic dysfunction. Combined with phase contrast imaging, morphologic assessment at MRI may enable grading of left ventricular diastolic function. Further work is ongoing to quantify relaxation on cine imaging using an automated algorithm.

